# Robotic-assisted partial nephrectomy for a neuroendocrine tumor in a horseshoe kidney: a case report

**DOI:** 10.1093/jscr/rjae292

**Published:** 2024-05-05

**Authors:** Kazuro Kikkawa, Kouhei Maruno, Tatsuya Hazama, Toshifumi Takahashi, Yuya Yamada, Masakazu Nakashima, Masahiro Tamaki, Noriyuki Ito

**Affiliations:** Department of Urology, Japanese Red Cross Wakayama Medical Center, 4-20, Komatsubaradori, Wakayama 640-8558, Japan; Department of Urology, Japanese Red Cross Wakayama Medical Center, 4-20, Komatsubaradori, Wakayama 640-8558, Japan; Department of Urology, Japanese Red Cross Wakayama Medical Center, 4-20, Komatsubaradori, Wakayama 640-8558, Japan; Department of Urology, Japanese Red Cross Wakayama Medical Center, 4-20, Komatsubaradori, Wakayama 640-8558, Japan; Department of Urology, Japanese Red Cross Wakayama Medical Center, 4-20, Komatsubaradori, Wakayama 640-8558, Japan; Department of Urology, Japanese Red Cross Wakayama Medical Center, 4-20, Komatsubaradori, Wakayama 640-8558, Japan; Department of Urology, Japanese Red Cross Wakayama Medical Center, 4-20, Komatsubaradori, Wakayama 640-8558, Japan; Department of Urology, Japanese Red Cross Wakayama Medical Center, 4-20, Komatsubaradori, Wakayama 640-8558, Japan

**Keywords:** neuroendocrine tumor, horseshoe kidney, robotic-assisted partial nephrectomy, transection of isthmus

## Abstract

Neuroendocrine tumors of the kidney are exceedingly rare. We report the first case of robotic-assisted partial nephrectomy for such tumors in horseshoe kidneys. A 65-year-old woman was incidentally found to have a 27 mm renal mass in the isthmus of her horseshoe kidney during computed tomography. Based on contrast-enhanced computed tomography results, we initially suspected renal cell carcinoma originating from the horseshoe kidney. Subsequently, robotic-assisted partial nephrectomy with isthmus transection was performed. Intraoperatively, we adjusted the port position for camera insertion and the patient’s positioning to facilitate better visualization for dorsal isthmus and vessel dissection. Pathological examination and immunohistochemical analysis revealed a well-differentiated neuroendocrine tumor. Therefore, robotic-assisted partial nephrectomy is a safe and effective approach for managing neuroendocrine tumors in the isthmus of horseshoe kidneys. Given the nonspecific clinical presentation of renal neuroendocrine tumors and their rarity, the optimal management of these tumors remains controversial.

## Introduction

Neuroendocrine tumors (NETs) are rarely observed in the kidney, although they can occur in multiple organs [1]. Most renal NETs arise in a normal kidney, but they may also manifest in a horseshoe kidney [2].

A horseshoe kidney is a congenital fusion anomaly, often accompanied by a complex blood supply [3]. Renal neoplasms occurring in horseshoe kidneys are infrequent, and performing minimally invasive surgery for renal tumors in these cases is technically challenging due to the variability of renal vessels [4].

To our knowledge, we present the first case of a patient with NET in the isthmus of a horseshoe kidney, managed through robotic-assisted partial nephrectomy (RAPN).

## Case report

**Figure 1:**
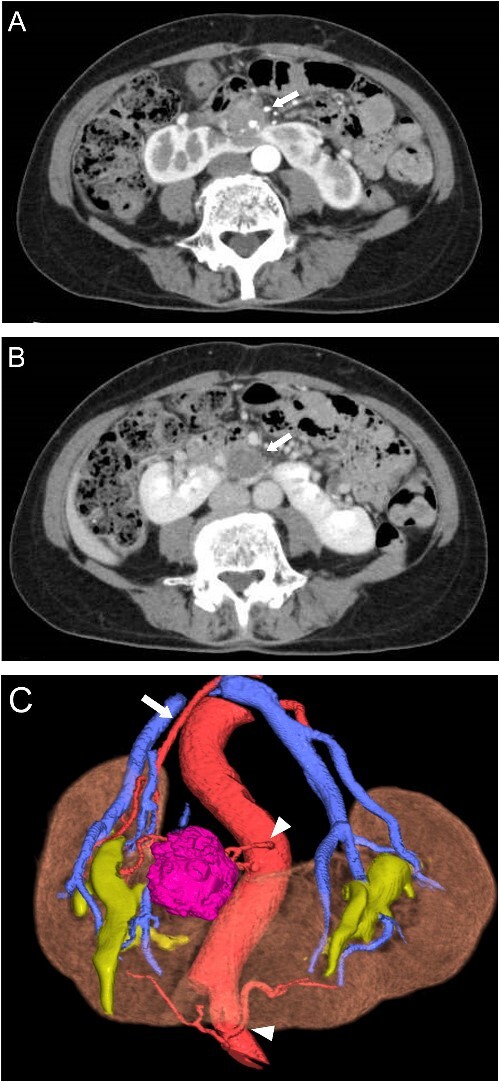
Preoperative CT scans. (A) Contrast-enhanced CT showed heterogeneous enhancement of the tumor in the arterial phases (arrow). (B) The cystic area of the tumor had no enhancement in the venous phase (arrow). (C) 3D-CT demonstrated one artery arising from the aorta to the right kidney (arrow) and two arteries supplying the isthmus and tumor arising from the ventral side of the aorta and left common iliac artery (arrowhead).

A 65-year-old woman presented to our department with an incidental 27 mm renal tumor in the isthmus of horseshoe kidney. Contrast-enhanced computed tomography (CT) imaging revealed that this tumor displayed slight enhancement with a solid component, accompanied by cystic and calcified elements (Fig. 1A and B). Based on these findings, we clinically diagnosed the renal cell carcinoma cT1aN0M0. Further assessment via three-dimensional (3D) CT revealed a single artery arising from the aorta to the right kidney, along with two arteries supplying the cephalic and caudal sides of the isthmus from the aorta and iliac artery, respectively (Fig. 1C).

**Figure 2:**
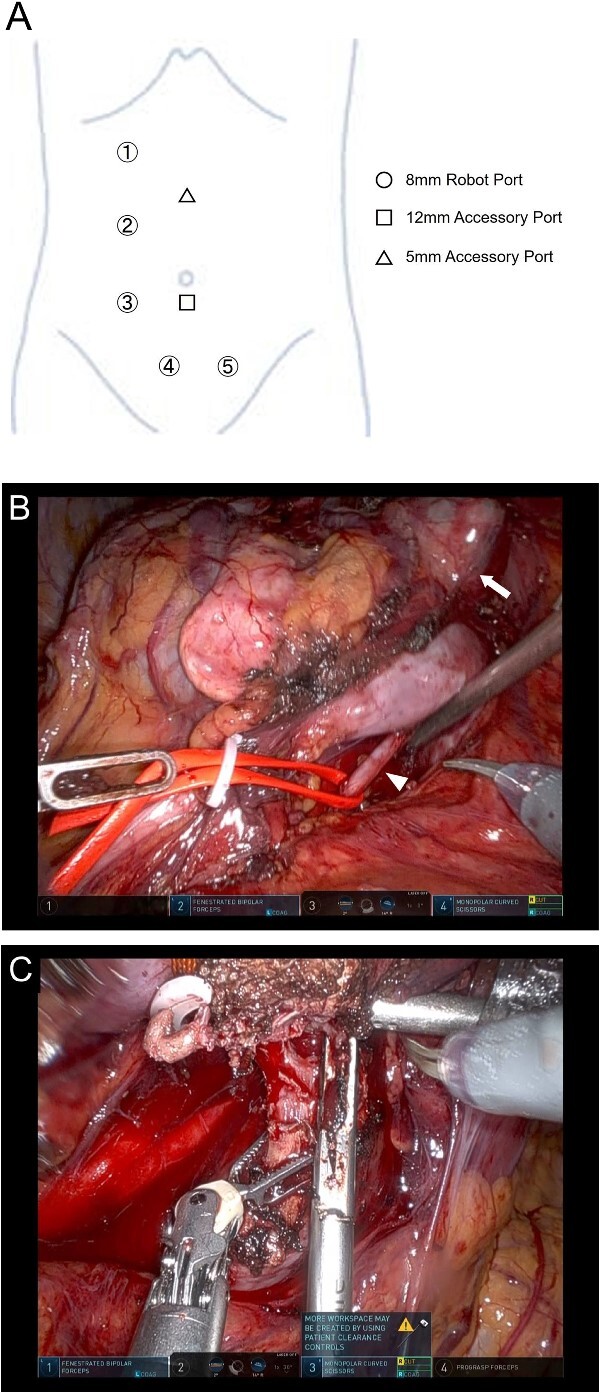
Position of the port for RAPN (A) and intraoperative findings of RAPN (B-D). (B) The dorsal side of the isthmus and artery were dissected with the number 4 port of the camera position. (C) The isthmus was transected with the number 3 port of the camera position.

Before RAPN, bilateral ureteral stents were placed. Five robotic ports and two assistant ports were inserted with the patient in the lateral position (Fig. 2A). Due to a history of abdominal surgery for uterine fibroids, appendicitis, and pelvic abscess, the intestine was extensively adhered to the lower abdominal wall. After securing the operating cavity through laparoscopic adhesiolysis, RAPN was initiated using the da Vinci Xi surgical system (Intuitive Surgical, Sunnyvale, CA, USA). Upon exposing the right renal artery, the caudal isthmus was not visible. Therefore, the position of the camera was switched to the port in the midline of the lower abdomen. Furthermore, by changing the patient’s position to semi-lateral and head-down, the caudal side of the isthmus became visible (Fig. 2B). After exposing the tumor and isthmus, two arteries supplying the cephalic and caudal sides of the isthmus were cut. Once the patient’s position was returned to the flank position and the camera position was switched to the outer port, the right renal artery was clamped, and the isthmus was transected using a vessel sealer (Fig. 2C). Subsequently, the tumor was resected with a sufficient margin.

**Figure 3:**
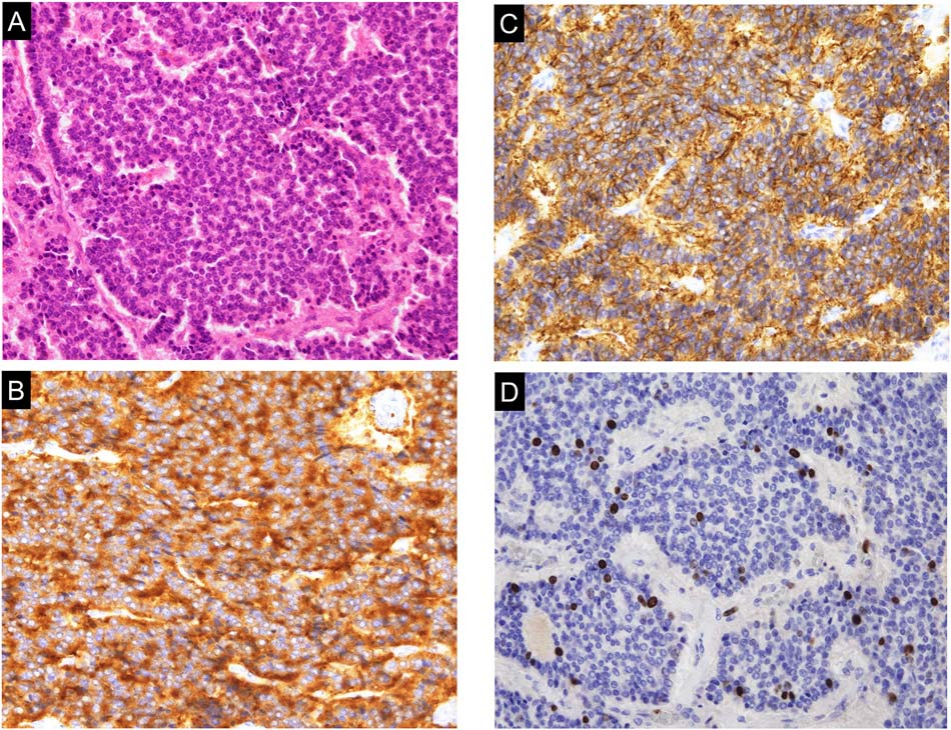
Histological and immunohistochemical examination of the specimen. A. The tumor was composed of small, round cells proliferating in a cord-like and ribbon-like papillary structure (hematoxylin and eosin staining; magnification, ×200). (B) The tumor cells were positive for synaptophysin (magnification, ×200). (C) The tumor cells were positive for CD56 (magnification, ×200). (D) The Ki-67 index was 4% (hematoxylin and eosin staining; magnification, ×200).

The operation lasted 355 minutes, with 196 minutes spent on the console. The warm ischemia time was 19 minutes. Histopathology revealed that the tumor consisted of small, round cells arranged in ribbon and cord-like structures (Fig. 3A). Immunohistochemistry analysis revealed positivity for synaptophysin (Fig. 3B), chromogranin A, CD56 (Fig. 3C), and vimentin, while CD10 and cytokeratin 7 were negative. Additionally, the Ki67 index was 4% (Fig. 3D). Based on these findings, the renal tumor was diagnosed as a well-differentiated NET. At the 6-month follow-up, no local recurrence or metastasis was detected in CT.

## Discussion

Renal NETs are rare, constituting only 0.18% of all primary renal neoplasms in a normal kidney [5]. However, the risk of developing NETs in a horseshoe kidney is 62-fold higher than in a normal kidney [2]. While common symptoms include abdominal pain, weight loss, and hematuria, ~25% of cases are diagnosed incidentally [6]. Conventional examinations do not reliably distinguish NETs from other renal tumors. NETs typically present as well-circumscribed and slightly enhanced masses on CT scans, often with a solid component occasionally accompanied by cystic and calcified components [7]. Histopathologically, tumor cells are arranged into cords and ribbons structures [8]. However, NETs lack typical organoid architecture, necessitating immunohistochemical analyses for diagnosis. NETs usually express at least one neuroendocrine marker, such as synaptophysin, chromogranin A, or CD56 [9], while CD10 and cytokeratin 7 are negative markers. Therefore, a combination of investigations for these markers is essential for an accurate diagnosis of renal NET. Furthermore, Ki67 serves as a reliable pathological grading marker according to the World Health Organization classification [6]. In our case, the Ki67 proliferation index was 4%, indicating a grade G2 NET. NETs are characterized by a low degree of malignancy and slow growth, making surgical radical resection the preferred approach. However, some cases may develop systemic metastases several years after resection, underscoring the necessity of long-term follow up [9].

Horseshoe kidney is a congenital renal fusion anomaly associated with multiple vascular changes, occurring in 0.15% to 0.25% of the population [3]. Preoperative imaging of blood vessels is necessary for surgery on renal tumors with horseshoe kidneys. Additionally, 3D CT is more useful for identifying various vasculatures [10]. Although there have been several reports of RAPN for renal tumors in horseshoe kidneys, there has been only one report of RAPN for renal tumor in the isthmus of a horseshoe kidney; in particular, isthmus transection was simultaneously performed via pure robotic surgery [11]. RAPN is suitable for small renal masses, particularly in cases involving horseshoe kidneys, owing to the advanced instrumentation of robotic systems in dissecting complex vessels and performing tumor resection, compared to conventional laparoscopic approaches. In our investigation, although the flank position and the usual camera position were optimal for accessing the renal artery, dissecting the dorsal side of the isthmus was challenging due to poor visibility. Consequently, we relocated the camera to the lower abdominal port, following the method described by Sawada *et al.* [11]. Additionally, adjusting the patient’s position to a semi-lateral, head-down orientation facilitated the safe transection of the dorsal vessels and isthmus. Therefore, by adapting camera and body positions, RAPN can be safely performed for intricate renal tumors near the isthmus.

Renal NET is a rare neoplasm, and precise diagnosis often hinges on immunohistochemical analysis. Performing minimally invasive surgery for renal tumors in horseshoe kidneys presents unique challenges due to anatomical anomalies. Establishing a preoperative surgical strategy, including a comprehensive evaluation of the location of tumor localization and vessel anatomy through 3D-CT imaging, is necessary to safely perform RAPN.

## Data Availability

The data underlying this article will be shared on reasonable request to the corresponding author.
